# Expression of *OsTPX* Gene Improves Cellular Redox Homeostasis and Photosynthesis Efficiency in *Synechococcus elongatus* PCC 7942

**DOI:** 10.3389/fpls.2018.01848

**Published:** 2018-12-11

**Authors:** Young-Saeng Kim, Jin-Ju Kim, Seong-Im Park, Spencer Diamond, Joseph S. Boyd, Arnaud Taton, Il-Sup Kim, James W. Golden, Ho-Sung Yoon

**Affiliations:** ^1^Research Institute of Ulleung-do and Dok-do, Kyungpook National University, Daegu, South Korea; ^2^Department of Biology, College of Natural Sciences, Kyungpook National University, Daegu, South Korea; ^3^School of Life Sciences, BK21 Plus KNU Creative BioResearch Group, Kyungpook National University, Daegu, South Korea; ^4^Division of Biological Sciences, San Diego, La Jolla, CA, United States; ^5^Advanced Bio-Resource Research Center, Kyungpook National University, Daegu, South Korea

**Keywords:** cyanobacteria, defense system, hydrogen peroxide, redox balance, thioredoxin peroxidase

## Abstract

Cyanobacterial 2-Cys peroxiredoxin (thioredoxin peroxidase, *TPX*) comprises a family of thiol antioxidant enzymes critically involved in cell survival under oxidative stress. In our previous study, a putative *TPX* was identified using a proteomics analysis of rice (*Oryza sativa* L. *japonica*, *OsTPX*) seedlings exposed to oxidative stress. This *OsTPX* gene is structurally similar to the *Synechococcus elongatus TPX* gene in the highly conserved redox-active disulfide bridge (Cys114, Cys236) and other highly conserved regions. In the present study, the *OsTPX* gene was cloned into rice plants and *S. elongatus* PCC 7942 strain to study hydrogen peroxide (H_2_O_2_) stress responses. The *OsTPX* gene expression was confirmed using semi-quantitative RT-PCR and western blot analysis. The *OsTPX* gene expression increased growth under oxidative stress by decreasing reactive oxygen species and malondialdehyde level. Additionally, the *OsTPX* gene expression in *S. elongatus* PCC 7942 (OT) strain exhibited a reduced loss of chlorophyll and enhanced photosynthesis efficiency under H_2_O_2_ stress, thereby increasing biomass yields twofold compared with that of the control wild type (WT) strain. Furthermore, redox balance, ion homeostasis, molecular chaperone, and photosynthetic systems showed upregulation of some genes in the OT strain than in the WT strain by RNA-Seq analysis. Thus, *OsTPX* gene expression enhances oxidative stress tolerance by increasing cell defense regulatory networks through the cellular redox homeostasis in the rice plants and *S. elongatus* PCC 7942.

## Introduction

The metabolism of most aerobic organisms inevitably produce reactive oxygen species (ROS) that are capable of irreversibly damaging vital cell components. In response to rising levels of ROS, cells employ additional strategies to limit damage (Day et al., 2012). For instance, signaling pathways are activated to increase the levels of detoxification and repair enzymes. Paradoxically, hydrogen peroxide (H_2_O_2_), one of the most representative ROS, is associated with this cellular signaling pathway. H_2_O_2_ concentrations increase in response to various abiotic and biotic stresses and take part in the reactive oxygen regulatory network (Mittler et al., 2004). Therefore, the intracellular H_2_O_2_ concentration needs to be carefully regulated to balance its toxic and signaling activities (Puerto et al., 2015).

To modulate peroxide balance, cells express this set of H_2_O_2_-decomposing enzymes; catalase peroxidase, ascorbate peroxidase, glutathione peroxidase, and 2-Cys peroxiredoxin (thioredoxin peroxidase, *TPX*). *TPX*, a thiol-specific peroxidase, catalyzes the reduction of H_2_O_2_ and plays a role in protecting cells against oxidative stress by detoxifying peroxides, and by acting as a sensor for hydrogen peroxide-mediated signaling events. Furthermore, *TPX* belongs to a conserved family of antioxidant genes, the peroxiredoxin, which use thiol groups as a source of reducing equivalents to scavenge oxidant species (Chae et al., 1994). The catalytic breakdown of H_2_O_2_ by *TPX* gene involves the oxidation of the peroxide-reactive peroxidatic cysteine (Cys), located in the N-terminal of proteins (Dietz et al., 2006), followed by the formation of a disulfide with the resolving Cys (Day et al., 2012). Subsequently, thioredoxin (TRX) reduces the oxidized TPX back to the monomeric form. These reactions are critical in protecting cells from ROS-induced stress and minimizing their subsequent degradation (Dietz, 2007).

A number of studies have addressed the function of *TPX* gene in peroxide detoxification and redox regulation under stress conditions in various species. For instance, *TPX* gene protects cells against H_2_O_2_ induced damage in humans (Berggren et al., 2001). *TPX* not being expressed resulted in weakened phenotypes that were very sensitive to oxidative stress within *Arabidopsis thaliana* (Finkemeier et al., 2005) and *Synechocystis* sp. PCC 6803 (Klughammer et al., 1998). Additionally, overexpression of heterologous *TPX* in transgenic plants conferred heat and methyl viologen tolerance in tall fescue (Kim et al., 2010), and promoted higher resistance against oxidative stress by enhancing antioxidant activity in transgenic tobacco (Lee et al., 2000). Moreover, our previous study demonstrated that expression of *Oryza sativa* (*O. Sativa*) *TPX (OsTPX)* increased oxidative stress tolerance and fermentation capacity by improving cellular redox homeostasis in *Saccharomyces cerevisiae* (Kim et al., 2013). The TPX sequences of the above-mentioned species have strictly conserved residues that function as redox active sites, which are functionally similar to conserved residues. These TPX sequences possess enhanced abilities to catalyze antioxidants and antioxidant-related enzymes under ROS-induced oxidative stress. Because the function of the redox active site is functionally similar to these conserved residues, heterologous *TPX* could act in a role similar to the cyanobacteria *TPX*. However, interaction with heterologous *TPX* has not been extensively studied in cyanobacteria under ROS-induced oxidative stress.

In the previous study, the heterologous expression of the *OsTPX* gene in the genetically modified *Synechococcus elongatus* (*S. elongatus*) strain PCC 7942 was found to improve tolerance to stress, photosynthetic efficiency, and cell viability under ROS-induced oxidative stress. Therefore, in the present study we profiled gene expression to identify the mechanisms preventing H_2_O_2_ stress. Our results demonstrated improved cellular redox homeostasis by enhancing oxidative stress tolerance and photosynthesis efficiency upon the introduction of the heterologous *OsTPX* gene, which affected the cell defense regulatory network through redox balance in *S. elongatus* PCC 7942.

## Materials and Methods

### Plant Growth Conditions

The *TPX* gene from *O. sativa* L. *japonica* was transfected into Ilmi cultivars of rice plants. Genotypes and phenotypes were screened by germinating transgenic rice plants and non-transgenic wild type (WT) rice seeds at 28°C for 3 days. The subsequently germinated seedlings were transplanted and grown for 4 weeks in a greenhouse (28–32°C, 16 h light/8 h dark cycle). The seedlings were then placed in a 50 mM H_2_O_2_ solution for 1 week each in three independent biological replicates and were used in the following experiments.

### Amino Acid Sequence Alignment

The BLAST software^1^ was used to align OsTPX with known TPX sequences using the National Center for Biotechnology Information (NCBI) database. The gene sequences were as follows: OsTPX, accession no. AK068919.1; SeTPX, accession no. AF492495.1; ScTPX, accession no. NC_001145.3; AnTPX, accession no. BA000019.2; MaTPX, accession no. AP009552.1; TeTPX, accession no. BA000039.2; AmTPX, accession no. CP000828.1; and AtTPX, accession no. AF324996.2, which has been shown to be a soluble monomeric enzyme that contains one molecule of 2-Cys per enzyme molecule, enabling the assessment of different amino acid positions present in protein active sites.

### Conjugation and Construction of Recombinant Plasmid *OsTPX*

The chloroplastic *2-Cys peroxiredoxin BAS1* (accession no. AK068919.1; *OsTPX*) coding region was amplified from cDNA using PCR with ExTaq polymerase (Takara Bio Inc., Shiga, Japan). The PCR reaction conditions were as follows; initial denaturation at 94°C for 3 min, followed by 30 cycles of 94°C for 30 s, 54°C for 30 s, 72°C for 1 min, and a final extension for 7 min at 72°C. The *OsTPX* gene was PCR cloned using sense and antisense primers, respectively (Supplementary Table S2). The PCR product was purified using a gel extraction kit (Nucleogen, Siheung, South Korea) and then inserted into the rice constitutive vector *pGA2897* and cyanobacterial expression vector *pCV0069* (Supplementary Table S1) (Kim et al., 2017). Competent *Escherichia coli* DH5α cells that had been transformed with the binary vectors were selected using hygromycin and nourseothricin (*Nat*, 50 μg mL^-1^). Expression clones were confirmed by sequencing before using them to transform *O. sativa* and *S. elongatus*. The binary vector of rice was introduced into *Agrobacterium* strain LBA4404 by electroporation and subsequent plant transformation in *O. sativa* (Hiei et al., 1994). The cloned cyanobacterial plasmid was sequenced using the NS2 primers (Supplementary Table S2), which were complementary to the neutral site II (NS2) region, to confirm proper gene ligation and direction. Finally, the resulting plasmid was assembled using the GeneArt Seamless Cloning and Assembly kit (Life Technologies, United States). The plasmids from cloned DNA were conjugated to *S. elongatus* using published protocols (Golden et al., 1987).

### Semi RT-PCR

The cDNA was reverse transcribed from total RNA using the SuperScript III kit (Life Technologies, United States). Semi-RT-PCR was performed as follows; one cycle at 95°C for 3 min, followed by 26–28 cycles at 94°C for 30 s, 54°C for 30 s, 72°C for 40 s, and then a final extension at 72°C for 5 min. The semi-RT-PCR amplicons were resolved using a 0.7% agarose gel in 0.5× Tris/borate/EDTA buffer. The primer sets are described in Supplementary Table S2. The *tubulin* and *rpoA* gene primer sets were used for the housekeeping control and were amplified.

### Western Blot Analysis

For the western blot analysis of the crude extracts, total cyanobacterial protein (OD_730_ = 0.30) was obtained using a suspension of a cold extraction buffer containing; 150 mM NaCl, 50 mM Tris-HCl, pH 7.3, 1 mM EDTA, 2% β-mercaptoethanol, 1 mM dithiothreitol, 1 mM phenylmethylsulfonyl fluoride, which were mixed with an equal volume of phenol saturated with Tris-HCl at pH 7.3. The rice protein was extracted from tissues using published protocols (Kim et al., 2013). Briefly, crude protein extracts were prepared using glass beads. Cells grown for 7 days were exposed to 2.5 mM H_2_O_2_ for 2 h to induce cellular oxidative stress, followed by vigorous vortexing of 10 times for 1 min on ice. The protein extracts were then cleared by centrifugation at 12,000 rpm for 20 min at 4°C. Finally, the protein concentrations were determined using a Pierce bicinchoninic acid protein assay kit (Thermo Scientific, Waltham, MA, United States) (Komatsu et al., 2009; Peng et al., 2011). Protein extracts (20 μg) were separated using 10% SDS-PAGE at 100 V and transferred onto polyvinylidene fluoride membranes (Bio-Rad, Hercules, CA, United States). These were then incubated in a blocking buffer consisting of 5% non-fat skim milk, and 0.02% sodium azide in Tris-buffered saline plus Tween (TBST, 10% Tween-20, 20 mM Tris-HCl, pH 7.6; and 150 mM NaCl), for 1.5 h at 24°C. The blots were then incubated overnight at 4°C with anti-TPX (Ab Frontier, Seoul, South Korea) antibodies appropriately diluted with a blocking buffer. The blots were then washed three times for 30 min with TBST, after which they were incubated with conjugated anti-rabbit secondary antibodies (Santa Cruz Biotechnology, Santa Cruz, CA, United States) diluted with a blocking buffer (without 0.02% sodium azide) for 4 h at 25–30°C. After washing with TBST, proteins binding to antibodies on the blots were visualized using the SuperSignal West Femto substrate kit (Pierce, Rockford, IL, United States) and imaged using a MultiImage II Light Cabinet (DE-500) (Alpha Innotech Corporation, San Leandro, CA, United States).

### Cyanobacterial Growth and Stress Treatment Condition

After culturing for 2 days at 30°C with shaking; aliquots of each sample were collected and the absorbance was measured at 730 nm. When the OD_730_ value was 0.3, the aliquots were diluted, and new measurements were taken. Cyanobacteria cells were cultured in BG11 medium containing 2.5 mM H_2_O_2_ for 7 days. To evaluate the response to oxidative stress, cells were allowed to reach the growth phase (OD_730_ = 0.30–0.35), exposed to 2.5 mM H_2_O_2_ for 2 days at 30°C with shaking. For plate cell-spotting assay, the OT, NS, and WT strains (OD_730_ = 0.35) were exposed to 2.5 mM H_2_O_2_ for 2 days and were serially diluted (10-fold) with distilled water. The diluted samples were then spotted on agar-solidified BG11 medium, followed by the cell suspensions being incubated for 2 days at 30°C before they were photographed or counted.

### Measurement of Ion Leakage

Ion leakage was analyzed using leaf disks, as described by Lee et al. (2007), with slight modifications. Ten rice leaf disks from five different plants were immediately floated on a solution containing 10 μM methyl viologen (MV) in deionized H_2_O. The leaf disks were incubated in dark for 12 h at 25°C to allow the MV to diffuse into the leaves. After pre-incubation, the leaf disks were placed under continuous white light until use. The extent of cellular membrane damage was quantified by ion leakage from 0 to 72 h using a conductivity meter (Isteck). At the end of the specified period, the samples were autoclaved for 15 min at 121°C. Next, the solution conductivity was measured again, and this value was considered 100% ion leakage in subsequent calculations of the relative ion leakage at different time periods. The visible damage caused by MV application was repeated three times.

### Measurement of MDA and ROS Level

The level of lipid peroxidation was determined in cells exposed to H_2_O_2_ for 2 days. The level of oxidative damage to lipids was determined by measuring the content of malondialdehyde (MDA) prepared in 10% trichloroacetic acid containing 0.65% 2-thiobarbituric acid (TBA) and heated at 95°C for 25 min. Finally, the MDA concentration of the resulting supernatant was measured at 532 nm, 600 nm, and estimated using an absorbance coefficient of 1.56 × 105, as described by Hodges et al. (1999). The level of lipid peroxidation was determined in rice plants by measuring MDA through a TBA assay (Kim et al., 2014). The intracellular H_2_O_2_ levels of cells exposed to H_2_O_2_ for 2 days were determined using the FOX reagent (100 μM xylenol orange, 250 μM ammonium ferrous sulfate, 100 mM sorbitol, and 25 mM sulfuric acid) by ferrous ion oxidation in the presence of a ferric ion indicator, xylenol orange (Kim et al., 2011). To measure the cellular ROS levels *in vivo*, strains harvested from the 100 mL culture (OD_730_ = 0.30–0.35) were exposed to 2.5 mM H_2_O_2_ for 2 days and incubated for 20 min at 30°C with 10 μM DCFHDA (Invitrogen) in the dark. Next, the cells were collected and analyzed using a fluorospectro-photometer (Cal Zeiss, LSM700) at an excitation of 488 nm and an emission wave length of 535 nm. Images were also captured by LSCM at 488 nm for excitation (Huang et al., 2012). The H_2_O_2_ levels in the rice plants were measured using the method described by Kim et al. (2014), with minor modifications.

### Measurement of Chlorophyll Content and Biomass Yield

For the chlorophyll content assays, strains were harvested from 100 mL cultures (OD_730_ = 0.30–0.35) after 2 days of 2.5 mM H_2_O_2_ treatment. The cultivated whole cells were harvested from 5 mL culture (OD_730_ = 0.3–0.35), with whole pellets being re-suspended in 90% methanol, and measured using the method of Halimatul et al. (2014) with some modifications. The absorption spectra of the cells were determined by harvesting 300 μL of the extracted chlorophyll in the presence and absence 2.5 mM H_2_O_2_. Next, the chlorophyll assays were performed by scanning from 260 to 800 nm using a spectrophotometer (Infinite M200 Pro microplate reader, Tecan). All assays had three biological replicates. For chlorophyll estimation within rice plants, the leaves were sampled from plants grown for 4 weeks in 50 mM H_2_O_2_ solution. Total chlorophyll contents were calculated using extraction coefficients and equations reported previously (Park et al., 2017), and expressed as values (%) relative to that of the WT rice plants (mg g^-1^ FW). The biomass yield was determined gravimetrically according to the method of Rai et al. (2014). A known volume of algal culture was centrifuged at 5,000 rpm for 10 min and the harvested biomass was vacuum-dried at 60°C until a constant weight was reached.

### Chlorophyll Fluorescence Image and Quantum Yield Analysis

Images were acquired using a FluorCAM 800MF, open version (PSI, Brno, Czech Republic) with a computer-operated control unit (SN-FC800-082, PSI) and a CCD camera (CCD381, PSI) with an F1.2 (2.8–6 mm) objective (Eneo, Japan). The data were analyzed according to Leal et al. (2015). An Aquapen-C AP-C 100 fluorometer (Photon Systems Instrument, Czechia) was used to measure chlorophyll fluorescence. The OJIP transient measurement induces fluorescence with blue plus red light, and detects fluorescence between 260 and 800 nm using bandpass filters (Kristoffersen et al., 2016).

### RNA-Seq Analysis

Three independent samples exposed to 2.5 mM H_2_O_2_ for 2 days were frozen with liquid nitrogen and ground using a mortar and pestle. Total RNA was isolated using TRIzol reagent (Invitrogen). Three biological replicates of each sample were used for RNA-Seq. RNA quality was assessed by an Agilent 2100 bioanalyzer using a RNA 6000 Nano Chip (Agilent Technologies, Amstelveen, Netherlands), and RNA quantification was made using ND-2000 Spectrophotometer (Thermo Inc., DE, United States).

For control and test RNAs, rRNA was removed from each 5 μg samples of total RNA using Ribo-Zero Magnetic kit (Epicenter, Inc., United States). Library construction was conducted using a SMARTer Stranded RNA-Seq Kit (Clontech lab Inc., CA, United States) according to the manufacturer’s instructions. First, a strand cDNA library is synthesized using a modified N6 primer. When the SMARTScribe Reverse Transcriptase reaches the 5′ end of the RNA fragment, the enzyme’s terminal transferase activity adds some additional nucleotides to the 3′ end of the cDNA. The carefully designed SMARTer Stranded Oligo base pairs with non-templated nucleotides, creating an extended template to enable the SMARTScribe RT to continue replication to the end of the oligonucleotide. The resulting full-length, single-stranded (ss) cDNA contains the complete 5′ end of the mRNA, as well as sequences that are complementary to the SMARTer Stranded Oligo. cDNA is generated to release the library from the beads, and the library is then amplified. Barcodes were introduced when the library was amplified. High throughput sequencing was performed as paired-end sequencing using HiSeq 2500 (Illumina, Inc., United States).

Bacterial-Seq reads were mapped using Bowtie2 software to obtain the alignment file. Differentially expressed genes were determined based on counts from unique and multiple alignments using EdgeR within R version 3.2.2 (R Development Core Team, e-biogen, South Korea) using BIOCONDUCTOR version 3.0 (Gentleman et al., 2004). The alignment file was used for assembling transcripts. A global normalization method was used for comparisons between samples. The sample was repeated three times and the fold change was calculated as the average value. Gene classification was based on searches performed using DAVID^2^. The GEO accession number is GSE122841.

### Statistical Analysis

All of the OT strain biochemical results were calculated relative to those of the WT and neutral-site vector (NS) strains grown under normal conditions, which were defined as 100%. Comparisons between individual data points were performed using Student’s *t*-test with a *P* < 0.05 being considered significant. All experiments were carried out at least in triplicate, and all results are expressed as the mean ± SD.

## Results

### *OsTPX* Gene Overexpressing Transgenic Rice Plants Improved H_2_O_2_ Stress Tolerance

**FIGURE 1:**
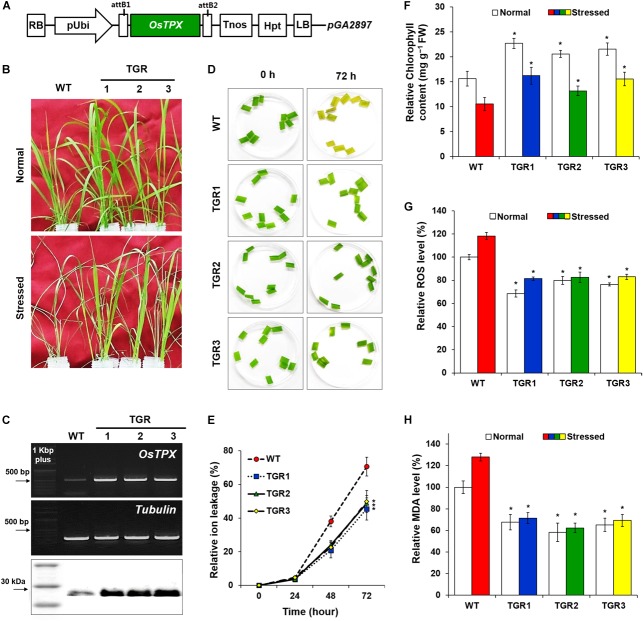
Tolerance of *OsTPX-*expressing transgenic rice under H_2_O_2_ stress. Rice seedlings were grown under greenhouse conditions (25–30°C) and experimental samples were obtained from 4-week old seedlings. **(A)** Schematic diagram of the *OsTPX* expression chimeric gene construct, *pUbi*::*OsTPX*. *OsTPX* gene is under the control of the maize ubiquitin promoter (*pUbi*), nos terminator (*Tnos*) and linked to the antibiotic resistance gene, hygromycin (*hgh*). LB, left border; RB, right border; attB1 and attB2, the Gateway recombination sites in *pGA2897* binary vector. **(B)** Stress phenotype of *OsTPX*-overexpressing transgenic rice (TGR) and wild-type (WT) plants in medium containing 50 mM H_2_O_2_ after 3 days. **(C)** Gene expression was determined using semi-qRT-PCR with *OsTPX* (245 bp) *tubulin* (220 bp) as the positive control and western blot with anti-TPX. One kb plus marker and protein color marker were used to determine band size. **(D,E)** Relative ion leakage was measured from leaf disks of rice plants floating in 10 μM MV solution. Leaf disks were incubated at 25°C for 72 h. The ion leakage percentages were calculated based on 100% of the values obtained after autoclaving. **(F)** Chlorophyll content, **(G)** ROS levels, and **(H)** MDA levels were determined as a measure of ROS scavenging ability. Error bars indicate ± SD of three independent experiments. Asterisks indicate significant differences between treatments as estimated by Student’s *t*-test (*P* < 0.05).

To facilitate the development of genetic engineering strategies to improve tolerance to abiotic stresses in plants, we previously identified the rice (*O. sativa* L. Ilmi) stress-responsive *OsTPX* gene and showed that it functions in yeast (Kim et al., 2013). To study the potential of *OsTPX* gene enhancing tolerance to other abiotic stresses such as H_2_O_2_, we produced transgenic rice plants expressing the *OsTPX* cDNA under the control of the maize ubiquitin promoter and nos terminator (Figure 1A). Under oxidative stress, the phenotype of *OsTPX* gene overexpression in transgenic rice (TGR) plants was superior to that of the WT (Figure 1B). Overexpression of the *OsTPX* gene was verified by detecting the mRNA and protein levels (Figure 1C). We examined ion leakage in leaves because ROS-induced stress disrupts cell membranes and releases cytoplasmic solutes. TGR plants were significantly less damaged than the control WT rice plants at each time interval following H_2_O_2_ treatment (Figures 1D,E). We also examined chlorophyll content, which was related to the photosynthetic ability of the leaf disks following oxidative stress treatment. As shown in Figure 1F, all the TGR plants exhibited higher chlorophyll content both with and without exposure to oxidative stress compared to that of the control WT plants. Moreover, both TGR and WT plants showed significantly increased ROS production under stress conditions, but the level of ROS was approximately 40% lower in TGR plants compared to the control WT plants (Figure 1G). To estimate the lipid peroxidation levels, the MDA content of TGR and WT plants were analyzed under H_2_O_2_ stress conditions. The increase in MDA content was more pronounced in control WT rice plants than it was in TGR rice plants (Figure 1H), suggesting that overexpressing *OsTPX* gene in TGR plants could protect the plant cell membrane from oxidative damage by enhancing ROS scavenging activity. These results showed that the *OsTPX* gene expression improved oxidative stress tolerance of the TGR plants.

### Amino Acid Sequence Alignment

**FIGURE 2:**
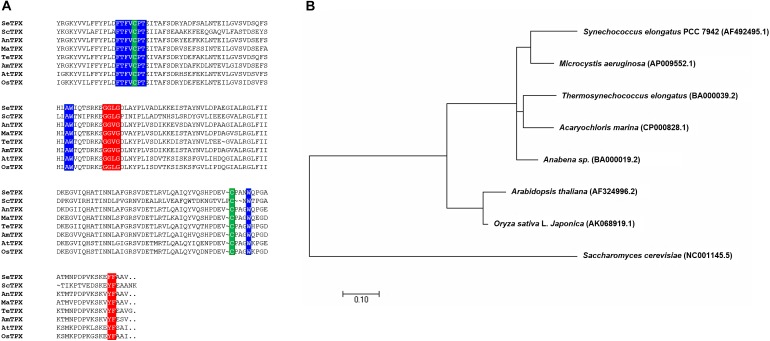
Alignment of TPXs. **(A)** Alignment showing highly conserved active site regions and highly conserved redox-active disulfide bridge cysteine. Backgrounds indicate: *TPX* active site regions with GGLG and YF (red backgrounds), FTFVCPT, AW, and W (blue backgrounds); cysteine residues involved in disulfide bridges (C and C; green backgrounds) **(B)** phylogenetic tree of *OsTPX* and other *TPX*s.

Multiple sequence alignment was performed to examine the amino acid sequence motifs of OsTPX, using known TPX sequences as described in the Materials and Methods. Pairwise alignment of OsTPX and other TPXs were conducted using BLAST software. OsTPX showed 73, 50, 74, 73, 76, 73, and 92% similarity to *S. elongatus* TPX (SeTPX), *Saccharomyces cerevisiae* TPX (ScTPX), *Anabaena* sp. TPX (AnTPX), *Microcystis aeruginosa* TPX (MaTPX), *Thermosynechococcus elongatus* TPX (TeTPX), *Acaryochloris marina* TPX (AmTPX), and *A. thaliana* TPX (AtTPX), respectively (Figure 2A). All of these sequences contain two conserved catalytic peroxidase cysteines, Cys114 and Cys236 (Figure 2A, green backgrounds), which likely form a disulfide bond with 2-Cys Prxs. *SeTPX* also possesses a highly conserved active-site region containing Thr111, Trp149, and Trp240 residues (FTFVCPT, AW, W; blue backgrounds) that are likely involved in peroxide reduction. Sequence alignment also shows the presence of *TPX* conserved motifs GGLG (Gly-Gly-Leu-Gly, red background) and YF (Tyr-Phe, red background), which are found in cyanobacteria but are absent in most prokaryotes (Pascual et al., 2010). Cyanobacterial *TPXs* contain the GGLG and YF conserved domains, which protect the active site from reacting with peroxides (Sayed and Williams, 2004; Pascual et al., 2010). These results show that the OsTPX protein contains the cysteines required for disulfide bond formation and the conserved active-site regions found in amino acid sequences of other TPXs. We constructed a phylogenetic tree containing the *SeTPX* (accession no. AF492495.1), which is 73% homologous to *OsTPX* (accession no. AK068919.1) (Figure 2B). The phylogenetic tree shows the evolutionary relationship between rice and cyanobacterial *TPX* genes. Thus, we selected the rice plant *OsTPX* gene to verify the function of the *TPX* gene for increasing cell defense regulatory networks through the cellular redox homeostasis in *S. elongatus* PCC 7942.

### Developing Genetically Transformed *OsTPX*-Expressing *S. elongatus* (OT) Strain

**FIGURE 3:**
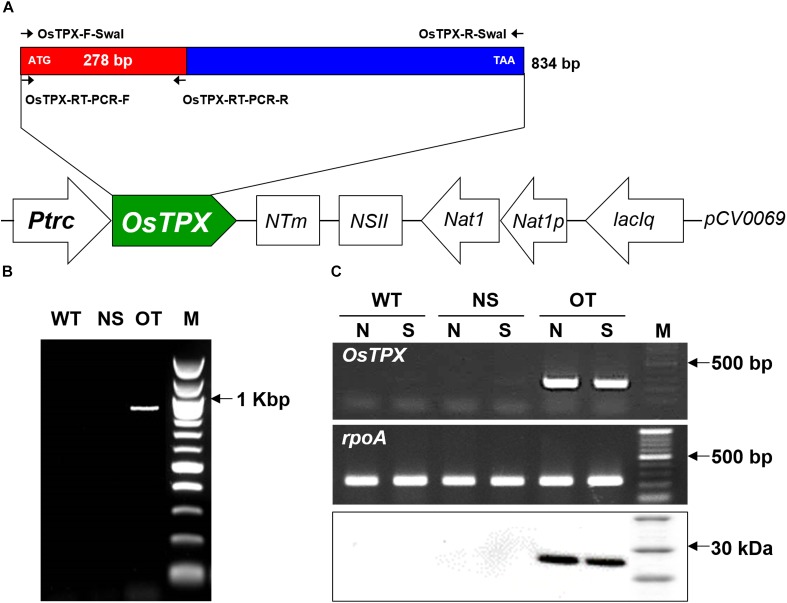
Construct of the OT strain and confirm gene expression. **(A)** Schematic diagram showing the expression of *OsTPX* in *S. elongatus*. *Ptrc*, IPTG-inducible promoter; *OsTPX*, *TPX* gene of *O. sativa*; *Nat*, nourseothricin antibiotic resistance; *lacIq*, lactose repressor. Arrows indicate direction of each gene. **(B)** PCR of *OsTPX* gene in transgenic *S. elongatus* (OT) to confirm genotype. **(C)** Expression of *OsTPX* gene was confirmed using semi-qRT-PCR (upper two panels) and western blotting (lower panel). The *rpoA* transcript was used as a control in the transcriptional analysis. N, normal condition; S, stressed condition.

An OT strain was constructed to evaluate H_2_O_2_-induced oxidative stress tolerance. The heterologous *OsTPX* gene was cloned into the neutral site II cyanobacterial expression vector *pCV0069* under the control of the IPTG-inducible promoter (*Ptrc*) (Figure 3A). Resistance to oxidative stresses was evaluated in cyanobacterial *S. elongatus* strains transformed with the *pCV0069*::*OsTPX* plasmid containing the heterologous *OsTPX* gene and the *pCV0069* vector alone. The genotypes were confirmed by PCR using primers flanking the *OsTPX* gene (Supplementary Table S2), which resulted in the expected fragment size of 834 bp (Figure 3B and Supplementary Table S1). To explore whether the *OsTPX* gene was effectively expressed, a semi quantitative RT-PCR analysis was performed using primers targeted inside the *OsTPX* gene (Supplementary Table S2), which resulted in the expected fragment size of 278 bp in the OT strain. However, no signal was detected in strains transformed with the empty NS and the WT strain (Figure 3C and Supplementary Table S1). We also performed western blot analysis to determine if the *OsTPX* gene was properly translated. The OT strain grown under H_2_O_2_ stress for 2 days showed the presence of a single band in the western blotting analysis using the plant anti-TPX antibody. The predicted molecular weight of the identified OsTPX protein was 28.079 kDa. No signal was detected in the NS and WT strains under the same conditions (Figure 3C). As a result, we transformed *S. elongates* with a transgene constructed from the heterologous *OsTPX* cDNA, regulated by a strong constitutive *Ptrc* promoter. These results indicate that the heterologous *OsTPX* gene and protein was expressed in the OT strain under control of the *Ptrc* promoter.

### An OT Strain Showed Tolerance to H_2_O_2_ Stress Condition

**FIGURE 4:**
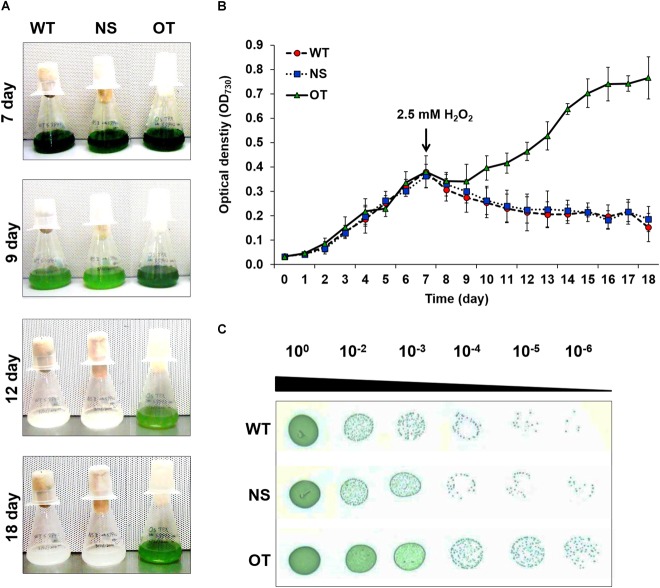
Analysis of WT, NS, and OT strains H_2_O_2_ stress tolerance. **(A)** Phenotypes, **(B)** growth phase of each strains, and **(C)** spotting assay and error bars calculated from the SD of three independent experiments. WT, wild-type; NS, empty neutral-site vector strain; OT, overexpression transgenic strain. Error bars indicate ± SD of three independent experiments. Asterisks indicate significant differences between treatments as estimated by Student’s *t*-test (*P* < 0.05).

Stress tolerance was measured using growth kinetics and colony formation on plates. IPTG (0.2 mM) was added to the NS and OT lines to activate the *Ptrc* promoter just before the stress treatment. The OT strain grew better than the NS and WT strains under H_2_O_2_ stress (Figures 4A,B). The NS and WT strains showed pigment losses on day 9, which was 2 days after exposure to H_2_O_2_ stress, and were completely bleached by day 12; whereas the OT strain showed relatively less pigment loss on days 9 and 12, and showed regrowth at 18 days (Figure 4B). As shown in Figures 4A,B, the OT strain containing the heterologous *OsTPX* gene showed significantly increased tolerance to H_2_O_2_ stress. Stress response was also determined using a plate cell-spotting assay. Exponentially growing OT, NS, and WT strains (OD_730_ = 0.30–0.35) were exposed to 2.5 mM H_2_O_2_ for 2 days and then serial dilutions were spotted on agar-solidified BG11 medium (Figure 4C). The OT strain showed higher cell viability than that of the NS and WT strains following exposure to high concentrations of H_2_O_2_. These results show that the heterologous *OsTPX* gene expression in the OT strain enhanced oxidative stress tolerance.

### Analysis of ROS Level and Cell Viability Under the H_2_O_2_ Stress

**FIGURE 5:**
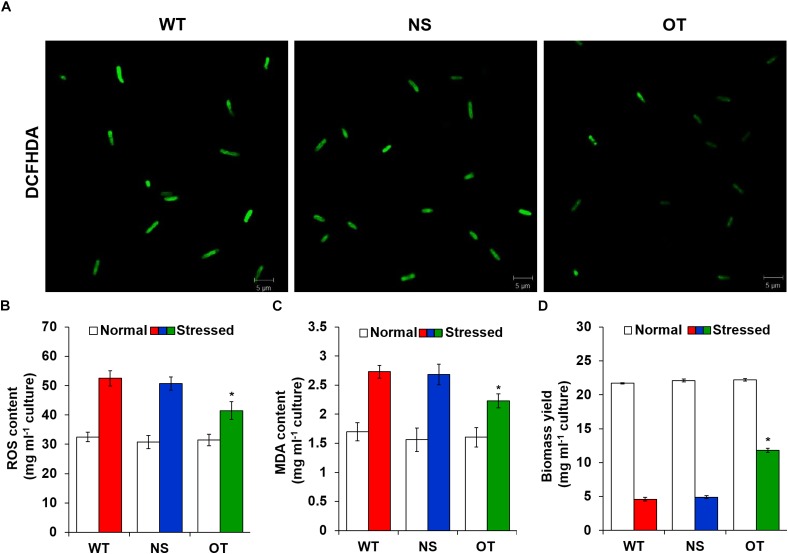
Measure ROS, MDA levels and dry weight under the oxidative stress. **(A)** Detection of DCFHDA fluorescence using confocal microscopy. **(B)** Intracellular ROS levels determined by FOX reagent. **(C)** The level of lipid peroxidation determined by MDA using a TBA assay. **(D)** Biomass yield measured after growth in the presence of high H_2_O_2_ concentrations. Error bars indicate SD of three independent experiments. Error bars indicate ± SD of three independent experiments. Asterisks indicate significant differences between treatments as estimated by Student’s *t*-test (*P* < 0.05).

To investigate the effects of oxidative stress produced by H_2_O_2_-generated ROS, ROS levels were analyzed *in vitro* and *in vivo* in the OT, NS, and WT strains. The redox state was determined based on the cellular ROS level assayed with the cytosolic oxidant-sensitive probe 2′,7′-dichlorodihydrofluorescein diacetate (DCFHDA), which measures the oxidative conversion of DCFHDA to the highly fluorescent compound dichlorofluorescein (DCF). NS and OT strains were added to 0.2 mM IPTG for *Ptrc* promoter activation just before exposure to H_2_O_2_. The three strains showed increased DCF fluorescence following exposure to 2.5 mM H_2_O_2_ for 2 days, but the fluorescence intensity of the probe was more pronounced in the control NS and WT strains than in the OT strain (Figure 5A). In a biochemical assay using the ferrous oxidation-xylenol orange (FOX) reagent, cellular ROS levels in the OT strain were approximately twofold lower than those in the NS and WT strains in the presence of H_2_O_2_ (Figure 5B). No differences were observed among the cells under normal conditions (Figure 5B, white columns). Furthermore, lower ROS production in the OT strain resulted in less MDA accumulation by lipid peroxidation compared to levels in the NS and WT strains (Figure 5C). MDA accumulation was 1.2-fold higher in NS and WT strains than in the OT strain. Additionally, the total biomass yield of the OT strain was 2.5-fold higher than that of the NS and WT strains subjected to oxidative stress (Figure 5D). Therefore, our results show that heterologous *OsTPX* gene expression in the OT strain conferred tolerance against oxidative stress high-yield biomass production compared to that observed in the control NS and WT strains.

### OT Strain Showed Improved Photosynthesis Capacity and Chlorophyll Content Under H_2_O_2_ Stress Conditions

**FIGURE 6:**
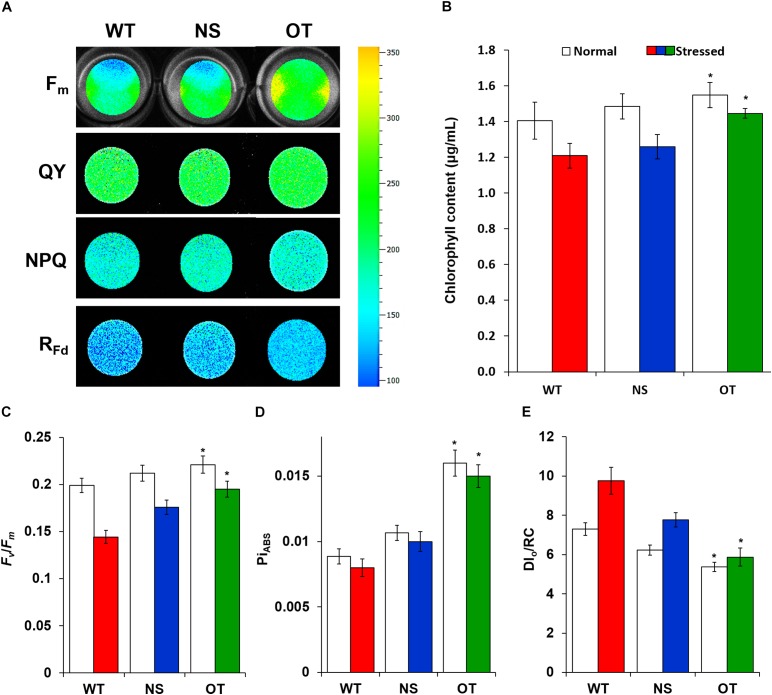
Comparison of chlorophyll fluorescence in cyanobacterial cells grown under normal and H_2_O_2_ stress conditions. **(A)** Fluorescence images of *F*_m_, QY, NPQ, and *R*_Fd_ in WT, NS, and OT strains. The higher efficiency rates are depicted in red and the lower rates in blue. **(B)** Chlorophyll content, **(C)** *F*_v_/*F*_m_, **(D)** Pi_ABS_, and **(E)** DI_0_/RC. *F*_m_, maximum fluorescence; QY, quantum yield of photochemical energy conversion in photosystem II; NPQ, non-photochemical quenching; *R*_Fd_, chlorophyll fluorescence decrease ratio; *F*_v_/*F*_m_, photochemical yield; Pi_ABS_, performance index for photosynthesis; DI_0_/RC, value of the energy dissipated per reaction center. Error bars indicate ± SD of three independent experiments. Asterisks indicate significant differences between treatments as estimated by Student’s *t*-test (*P* < 0.05).

All indicators of photosynthetic capacity were enhanced in the OT strain in the presence of H_2_O_2_ compared to those in the control NS and WT strains (Figure 6). NS and OT strains were added to 0.2 mM IPTG for *Ptrc* promoter activation just before H_2_O_2_ stress exposure. The major chlorophyll fluorescence parameters measured (Gorbe and Calatayud, 2012) were; maximal chlorophyll fluorescence intensity (*F*_m_), effective quantum yield of photochemical energy conversion in photosystem II (QY), non-photochemical quenching (NPQ), and chlorophyll fluorescence decrease ratio (*R*_Fd_) (Figure 6A). These fluorescence parameters and ratios were measured in a dark-adapted state to determine the functionality of photosystem II. For all parameters, the OT strain showed higher levels compared to those observed in the NS and WT strains (Figure 6A). Additionally, measurement of chlorophyll content showed smaller loss of chlorophyll in the OT strain than in the NS and WT strains under normal and stress conditions (Figure 6B). Variable to maximum fluorescence (*F*_v_*/F*_m_, photochemical yield), performance index for photosynthesis (Pi_ABS_), and dissipation energy per active reaction center (DI_0_/RC), were three indicators measured under the treated and untreated H_2_O_2_ conditions to provide a quantitative analysis of photosynthetic properties (Figures 6C–E). In particular, the *F*_v_*/F*_m_ ratio was significantly increased in the OT strain (Figure 6C) compared to that in the control strains. Additionally, Pi_ABS_, which is the performance index for energy conservation from photons absorbed by the PSII antenna until the reduction of PSI acceptors, was enhanced in the OT strain (Figure 6D). The DI_0_/RC ratio declined in the OT strain, showing that less absorbed light energy was dissipated as fluorescence (Figure 6E), which indicates a better photosynthetic efficiency compared to the NS and WT strains under normal and H_2_O_2_ stress conditions (Figure 6).

### Effect of *OsTPX* Gene Expression on the Cyanobacterial Antioxidant Defense System

**FIGURE 7:**
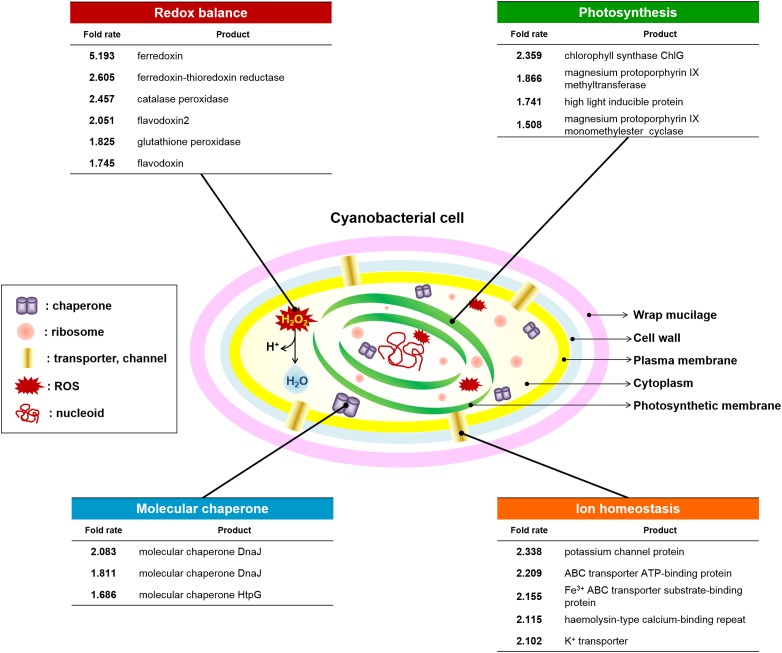
Changes in cyanobacterial defense system against ROS-induced oxidative stress. Redox balance (six genes), photosynthesis (four genes), Molecular chaperone (three genes), and ion homeostasis (five genes) were upregulated in OT strain under the H_2_O_2_ stress compared to the WT. Statistically significant changes indicate a fold-change, provided by e-biogen RNA-Seq Service Company (http://www.e-biogen.com). Detailed data about RNA-Seq analysis are presented in Supplementary Tables S5.

The OT strain exhibited improved intracellular ROS levels, redox homeostasis, photosynthetic ability, membrane stability, and growth during cultivation in the presence of H_2_O_2_. Therefore, we profiled gene expression to identify the mechanisms underlying *OsTPX*-mediated tolerance against H_2_O_2_ stress. OT strains were added to 0.2 mM IPTG for *Ptrc* promoter activation just before H_2_O_2_ stress exposure. RNA-sequencing (RNA-Seq) data identified 18 genes that were upregulated in the OT strain after treatment with 2.5 mM H_2_O_2_ for 2 days (Figure 7). All gene fold results were calculated as OT/WT fold. The results showed that expression of *OsTPX* gene increased redox balance (ferredoxin, 5.193; ferredoxin-thioredoxin reductase, 2.605; catalase peroxidase, 2.457; flavodoxin2, 2.051; glutathione peroxidase, 1.825; flavodoxin, 1.745), photosynthesis (chlorophyll synthase ChlG, 2.359; magnesium protoporphyrin IX methyltransferase, 1.866; high light inducible protein, 1.741; magnesium-protoporphyrin IX monomethyl ester cyclase, 1.508), ion homeostasis (potassium channel protein, 2.338; ABC transporter ATP-binding protein, 2.209; Fe^3+^ ABC transporter substrate-binding protein; hemolysin-type calcium-binding repeat, 2.115; K^+^ transporter, 2.102), and molecular chaperone (molecular chaperone DnaJ, 2.083; molecular chaperone DnaJ, 1.811; molecular chaperone HtpG, 1.686) in the OT strain compared to the WT strain. These results suggest that heterologous *OsTPX* gene expression in *S. elongatus* PCC 7942 improved cell defense regulatory networks from oxidative damage, and maintained the balance of cellular redox and photosynthesis.

## Discussion

Many studies have been performed to examine the effect of *TPX* gene overexpression on cellular metabolism for a wide variety of organisms (Chae et al., 1994; Yamamoto et al., 1999; Berggren et al., 2001; Broin and Rey, 2003). In previous studies, analysis of transgenic *Arabidopsis* with reduced *TPX* amounts demonstrated that the *TPX* protects chloroplast proteins from oxidative damage. Partial suppression of *TPX* expression caused impairment of photosynthesis and increased oxidative damage of chloroplast proteins during early plant development (Dietz et al., 2002). Similar results were found in the present study, where developed TGR plants overexpressed the *OsTPX* gene under the control of maize ubiquitin promoter (Figure 1A), confirming that the expression was at the mRNA and protein levels (Figure 1C). TGR plants conferred acquired H_2_O_2_ stress tolerance (Figure 1B) by improving higher cell stability and chlorophyll content (Figures 1D–F). Additionally, maintaining low MDA and ROS levels in rice plant cells (Figures 1G,H) indicated that TGR plants had an enhanced stress response than did the WT plants. However, the role of *TPX* gene in oxidative stress has not been studied in *S. elongatus* PCC 7942. To confirm the above results with other strains, including *S. elongatus* PCC 7942, the current study was conducted to determine if the expression of the *OsTPX* gene in *S. elongatus* enhanced its tolerance to H_2_O_2_-induced oxidative stress.

The enhancement of stress tolerance in the OT strain may be due to the heterologous *OsTPX* gene having similar functions through the highly conserved active regions of the protein sequences of OsTPX and cyanobacterial TPX. Interestingly, amino acid sequence alignment indicated that OsTPX shares a highly conserved active-site region; described green backgrounds (C), red backgrounds (GGLG, AW), and blue backgrounds (FTFVCPT, AW, W) in Figure 2A (Wood et al., 2003; Sayed and Williams, 2004; Moon et al., 2005; Pascual et al., 2010; Kim et al., 2013). Two catalytic cysteine residues (Cys 114 and Cys 236) of TPX could have important roles in neutralizing toxic ROS, which is consistent with a previous study that found human TPX*s* were involved in detoxifying ROS and protected cells from oxidative stress (Schröder et al., 2000). The susceptibility to over-oxidation has been considered specific for TPX and depends on the presence of two motifs, GGLG and YF, of these proteins (Wood et al., 2003). Additionally, modification of Thr66, Trp99, and Trp89 of barley HvTPX (Thr116 in FTFVCPT, Trp149 in AW, and Trp240 is W of OsTPX) involved these residues in the conformational change (dimer to oligomer) that altered protein function (König et al., 2003). Notably, the previous study showed that other *TPX*-mutated strains exhibited decreased TPX activity, indicating that these residues are essential for the catalysis of Thr48 of poplar PtTPX (Thr111 in OsTPX), and Cys64 of barley HvTPX (Cys114 of OsTPX) (König et al., 2003; Rouhier and Jacquot, 2008). The phylogenetic tree also shows the evolutionary relationship between rice and cyanobacterial *TPX* genes (Figure 2B). Therefore, these highly conserved regions play a key role in *TPX* gene function. It can be expected that the *OsTPX* gene of rice plants functions in a cooperative role in the oxidative stress tolerance that has been observed in cyanobacteria.

To investigate whether the expression of the *OsTPX* gene affects oxidative stress in cyanobacteria, we transformed *S. elongatus* PCC 7942 with the *OsTPX* gene under the control of the *Ptrc* promoter, and confirmed its expression at the mRNA and protein levels (Figures 3A,C). Our results show that heterologous *OsTPX* gene expression can enhance tolerance of cyanobacteria to oxidative stress such as H_2_O_2_ (Figure 4). It has been reported that the heterologous *TPX* gene is able to improve stress tolerance in many organisms (Lee et al., 2000; Jing et al., 2006). This may explain why the OT strain showed better growth than the control NS and WT strains did in the presence of abiotic stresses, including major ROS-generating agents, such as; 200 mM NaCl, a low 10°C temperature, 250 μE high-intensity light, and 20% sodium dodecyl sulfate (SDS) (Supplementary Figure S1). Previous studies found that the *TPX* gene increased salt and low temperature tolerance in *Arabidopsis* (Jing et al., 2006); and that the loss of *TPX* gene function in *Synechocystis* sp. resulted in significantly late grow rates compared to control wild-type strains under high light conditions (Kobayashi et al., 2004). In other studies, the *TPX* gene functioning as a chaperone indicated that it was effective in preventing protein aggregation induced by SDS stress (Jang et al., 2004; Messina et al., 2014). Taken together, heterologous *OsTPX* gene expression enhanced tolerance to abiotic stresses by eliminating ROS-induced oxidative stress.

As mentioned above, regulating H_2_O_2_ balance is very important to redox homeostasis in cell peroxidation. To investigate the effects of oxidative stress such as H_2_O_2_-generated ROS, ROS levels were measured *in vitro* and *in vivo* in the WT, NS, and OT strains (Figures 5A,B). Low ROS production in the OT strain resulted in less MDA accumulation by lipid peroxidation compared to that observed in the NS and WT strains. Therefore, the total biomass yield of the OT strain was decreased to a smaller extent upon exposure to oxidative stress (Figures 5C,D). These findings are consistent with a previous observation that the *TPX* gene is related to oxidative stress and could eliminate cellular damage caused by ROS in cyanobacteria (Klughammer et al., 1998; Stork et al., 2009; Pascual et al., 2010). Additionally, a gene disruption study showed that the *TPX* gene is essential for survival under oxidative stress conditions by maintain cell stability (Kobayashi et al., 2004). Thus, it can be speculated that heterologous *OsTPX* gene enhanced stress tolerance and biomass yield by preventing cell damage under the oxidative stress within *S. elongatus* PCC 7942.

Published studies have provided evidence of the relationship between stress tolerance and photosynthetic capacity, which is affected by *TPX* gene overexpression in several organisms (Nikkanen and Rintamaki, 2014; Brzezowski et al., 2015; Puerto et al., 2015; Guevara-Flores et al., 2017). For example, *TPX* gene expression conferred tolerance to long-day photoperiod and oxidative stresses by reducing the cellular ROS level in *Debaryomyces hansenii* (Chao et al., 2009) and *Pichia methanolica* (Puerto et al., 2015). We also found that the OT strain exhibited higher chlorophyll content (Figure 6B) and *F*_v_/*F*_m_ ratio under normal and H_2_O_2_ stress conditions (Figure 6C), and also showed an increased photosynthetic efficiency based on the QY, NPQ, *R*_Fd_, and Pi_ABS_ (Figures 6A,D). In contrast, the OT strain showed decreased DI_0_/RC values (Figure 6E), whereas the NS and WT strains exhibited considerably higher levels of energy dissipation than the OT strain under normal and 2.5 mM H_2_O_2_ conditions. This phenomenon is assumed to be related to an increase in gene expression that involved photosynthetic metabolism (Figure 7 and Supplementary Table S3). A high-light inducible gene, perhaps induced by *OsTPX* gene expression, could play a critical role in the adaptation of cyanobacterium through an increase in chlorophyll, and by improving the repair of the photosynthetic apparatus (Zeng et al., 2002; Havaux et al., 2003). Furthermore, genes related to the chlorophyll biosynthesis pathway were increased more in the OT strain than the WT strain. Several genes such as chlorophyll synthase ChlG, magnesium protoporphyrin IX methyltransferase, and magnesium protoporphyrin IX monoethyl ester cyclase are involved in tetrapyrrole biosynthesis pathway, which is known as the chlorophyll biosynthesis pathway in plants (Brzezowski et al., 2015). Therefore, these results indicated that heterologous *OsTPX* gene expression induced chlorophyll biosynthesis, resulting in enhanced photosynthetic capacity in *S. elongatus* PCC 7942.

Reduced TRX also reduces the oxidized TPX by ROS-induced stress. These reactions are critical in protecting cells under oxidative stress conditions (Dietz, 2007). Our results, which included gene profiling of WT and OT strains under H_2_O_2_ stress, showed an increase in antioxidant genes such as catalase peroxidase and glutathione peroxidase compared with control strains (Figure 7 and Supplementary Tables S3, S5). In addition, electron shuttles harboring iron–sulfur clusters such as ferredoxin, ferredoxin-thioredoxin reductase, and flavodoxin were increased in the OT strain. Ferredoxin is an important molecule involved in the routes of cyclic electron flow that operate under physiological and stress conditions, and is also used as a reducing equivalent by TRX and ferredoxin-thioredoxin reductase (Tognetti et al., 2006). In cyanobacteria, stress situations induce the synthesis of flavodoxin (Falk et al., 1995), an electron carrier flavoprotein that is not found in plants. The overexpression of flavodoxin in *E. coli* leads to an augmented tolerance toward various sources of oxidative stress (Zheng et al., 1999). These electron shuttles could be expected to maintain cellular homeostasis by regulating redox balance during electron transfer. However, those antioxidant and electron carrier genes show no differential expression levels between WT and OT strains under normal conditions (Supplementary Table S4). Interestingly, our results appear to show the level of cyanobacterial TRX (*SeTPX*) is more similar to the OT strain, than to the WT strain under the normal and stress conditions (Supplementary Table S4). This phenomenon assumed that *OsTPX* is not affected by *SeTPX* under normal and stress conditions; however, the expression of related genes were upregulated in the presence of H_2_O_2_. These results indicated that the heterologous *OsTPX* gene affected higher expression levels of antioxidant and electron carrier genes under the H_2_O_2_ stress conditions. Consequently, the OT strain improved cell homeostasis by maintaining a redox balance when compared to the WT strain.

Taken together, these findings indicate that the expression of the *OsTPX* gene in rice plants and *S. elongatus* resulted in an improvement in stress tolerance to ROS-induced oxidative stress and enhanced photosynthetic ability. In addition, cell membrane stability and biomass yield increased in the OT strain when compared to the WT and NS strains. These effects were achieved by enhancing the balance of cellular redox homeostasis through the activity of heterologous *OsTPX* gene expression, which resulted in the expression of related genes in the redox balance, photosynthesis, ion homeostasis, and molecular chaperone. In conclusion, our results found that *OsTPX* gene expression of rice plants could improve the cell defense regulatory network through cellular redox homeostasis under the oxidative stress in *S. elongatus* PCC 7942. Therefore, future studies on the genetic and molecular functions of the *OsTPX* gene should focus on the interactions between plants and cyanobacteria, and their responses to various environmental conditions.

## Author Contributions

Y-SK, I-SK, JG, and H-SY designed the project and were involved in the writing of the manuscript. Y-SK and J-JK wrote the manuscript. Y-SK and S-IP carried out most of the experiments and analyzed the data. SD, JB, and AT participated in the discussion and critiqued the writing.

## Conflict of Interest Statement

The authors declare that the research was conducted in the absence of any commercial or financial relationships that could be construed as a potential conflict of interest.

## Footnotes

1 http://clustalw.ddbj.nig.ac.jp/

2 http://david.abcc.ncifcrf.gov/
