# Supplementary material for: Expression of OsTPX Gene Improves Cellular Redox Homeostasis and Photosynthesis Efficiency in Synechococcus elongatus PCC 7942
Source: Front Plant Sci. 2018 Dec 11;9:1848. doi: 10.3389/fpls.2018.01848 (PMC6297720; doi:10.3389/fpls.2018.01848)
Supplement: Supplementary file 2 [file Data_Sheet_1.pdf]

Supplementary Materials for

Expression of *OsTPX* Gene Improves Cellular Redox Homeostasis and Photosynthesis Efficiency in *Synechococcus elongatus* PCC 7942

Young-Saeng Kim[1,†], Jin-Ju Kim[2,3,†], Seong-Im Park[2,3], Spencer Diamond[5], Joseph S. Boyd[5],

Arnaud Taton[5], Il-Sup Kim[4,*], James W. Golden[5,*], and Ho-Sung Yoon[2,3,4,*]

[1]Research Institute of Ulleung-do and Dok-do, [2]Department of Biology, College of Natural Sciences, [3]School of Life Sciences, BK21 Plus KNU Creative BioResearch Group, [4]Advanced Bio-resource Research Center, Kyungpook National University, Daegu 41566, Korea; [5]Division of Biological Sciences, University of California San Diego, La Jolla, California 92093, USA

Corresponding author. Email: Il-Sup Kim: 92kis@hanmail.net;

James W. Golden: jwgolden@ucsd.edu; Ho-Sung Yoon: hsy@knu.ac.kr

Supplementary Figure S1

Supplementary Tables S1 to S4

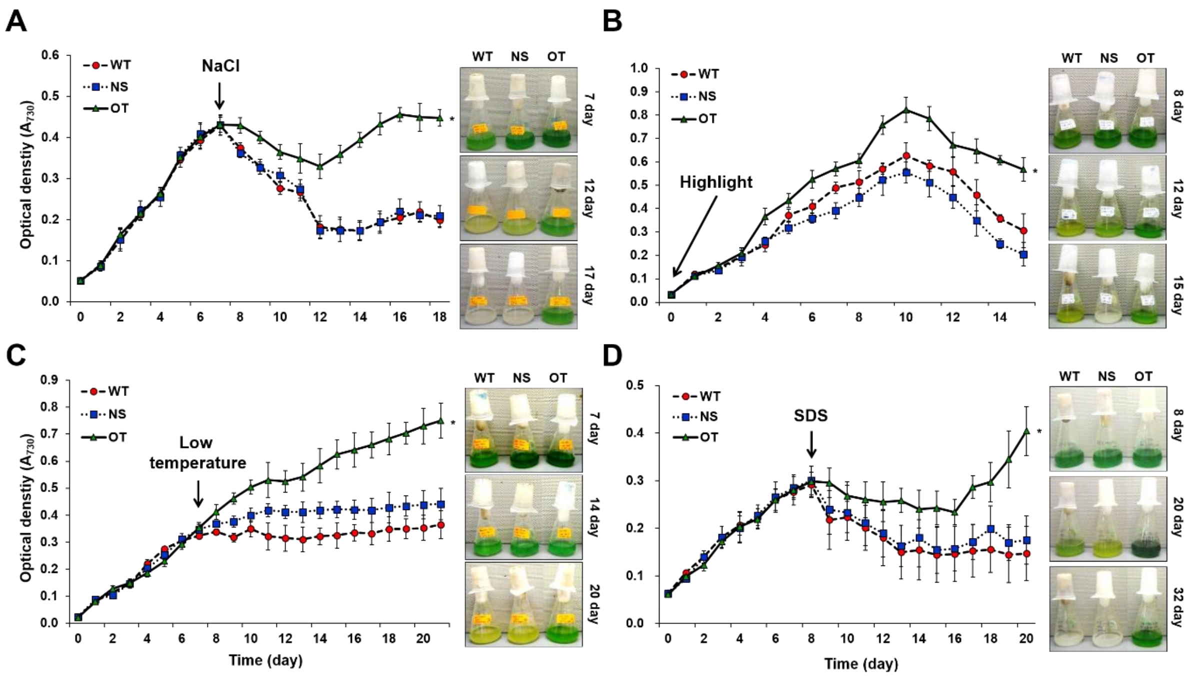

Supplementary Figure S1. Growth curve measurement of WT, NS, and OT strains under various abiotic stresses. The OT strain grows better than other strains under various stress conditions. (**A**–**D**) Strains were grown in only BG11 medium, and the initial optical density at 730 nm was adjusted to 3.0. Next, each sample was exposed to the following stress treatments: (**A**) 200 mM NaCl after 7 days, (**B**) 250 µE high light intensity during the first day, (**C**) 10 °C low temperature after 7 days, and (**D**) 20% SDS after 8 days. After culturing at 30 °C with shaking, aliquots of each sample were collected daily and absorbance was measured at 730 nm. Error bars indicate ± SD of three independent experiments. Asterisks indicate significant differences between treatments as estimated by Student's *t*-test (P<0.05).

Supplementary Table S1. Cyanobacterial strains and plasmids used in this study

Strain or Plasmid	Characteristic(s)	Antibiotic resistance	Source or reference
**Strains**			
AMC06	Wild type *Synechococcus elongatus* PCC 7942	none	Lab collection
DH5α	*Escherichia coli* cloning host	none	Lab collection
**Plasmids**	Relevant characteristics*		
pGA2897::OsTPX	The *pGA2897* binary vector plasmids that contained the maize ubiquitin promoter(*Ubi*) and *OsTPX* into the *A. tumefaciens* strain *LBA4404* via electroporation; Hpt^r	Hpt	This work
pCV0069	*S. elongatus* carrying *pCV0069* as a single recombinant; Nat^r	Nat	Lab collection
pCV0069::OsTPX	S7942NS2-Nat1-Ptrc/SwaI-TPX-RBS_OsTPX, 5815 bp, Nat^r , for IPTG-inducible *OsTPX* gene expression	Nat	This work

* Abbreviations for antibiotics: nourseothricin (Nat) and hygromycin B phosphotransferase (Hpt).

Supplementary Table S2. Primer sequences for PCR amplification

	Name	Sequence
O. sativa	OsTPX-F	CACCAATCCCCTCCTCATCCACT
	OsTPX-R	AAAAACTCCCTACATCCATCACC
	Tubulin-F	GAGTACCCTGACCGCATGAT
	Tubulin-R	GTGGTCAGCTTGAGAGTCCT
	OsTPX-RT-F	GCCGCTCCCACCGCAGCAGC
	OsTPX-RT-R	GGGCTTGTCGGAGAGGTCCATC
S. elongatus	rpoA-F	GCCGCCTGCTGCTCCTCCCT
	rpoA-R	AGCAAGTCAGCAACGGAGTTC
	NS2-F	GTCAACGTAAATGCATGCCGC
	NS2-R	GGTCACTACTTGGTAGCAACTC
	OsTPX-RT-PCR-F	ATGGCCGCCTGCTGCTCCTCCCT
	OsTPX-RT-PCR-R	CTGTCGAAGTGCAACTACTTGAGGACCAGC
	OsTPX-F-SwaI	TGCGGATCCTAAGGAGGAAAATTTATGGCCGCCTGCTGCTCCTCCCTCGCCACCGCCG
	OsTPX-R-SwaI	GCCGGGGAGCTCCTTCATTTATTTTTAGATGGCCGCGAAGTACTCCTTGCTTCCCTTGGG

Supplementary Table S3. **Upregulated genes expression levels under H_2O_2 stress screened using RNA seq**

Gene symbol	Fold change	Protein ID	Product
**Redox balance**			
SYNPCC7942_RS13385	**5.193**	WP_040113194.1	ferredoxin
SYNPCC7942_RS05790	**2.605**	WP_011377875.1	ferredoxin-thioredoxin reductase
SYNPCC7942_RS08440	**2.457**	WP_011244741.1	catalase peroxidase
SYNPCC7942_RS07035	**2.051**	WP_011378008.1	flavodoxin2
SYNPCC7942_RS02235	**1.825**	WP_011377579.1	glutathione peroxidase
SYNPCC7942_RS13770	**1.745**	WP_011242314.1	flavodoxin
**Photosynthesis**			
SYNPCC7942_RS10560	**2.359**	WP_011378308.1	chlorophyll synthase ChlG
SYNPCC7942_RS02240	**1.866**	WP_011243390.1	magnesium protoporphyrin IX methyltransferase
SYNPCC7942_RS05735	**1.741**	WP_071818114.1	high light inducible protein
SYNPCC7942_RS09665	**1.508**	WP_011244498.1	magnesium-protoporphyrin IX monomethyl ester cyclase
**Ion homeostasis**			
SYNPCC7942_RS08465	**2.338**	WP_011244737.1	potassium channel protein
SYNPCC7942_RS05795	**2.209**	WP_011242728.1	ABC transporter ATP-binding protein
SYNPCC7942_RS07205	**2.155**	WP_011242460.1	Fe^{3+} ABC transporter substrate-binding protein
SYNPCC7942_RS05775	**2.115**	WP_011377873.1	haemolysin-type calcium-binding repeat
SYNPCC7942_RS05545	**2.102**	WP_011242780.1	K^+ transporter
**Molecular chaperone**			
SYNPCC7942_RS11715	**2.083**	WP_011378399.1	molecular chaperone DnaJ
SYNPCC7942_RS12135	**1.811**	WP_071818122.1	molecular chaperone DnaJ
SYNPCC7942_RS09200	**1.686**	WP_011244591.1	molecular chaperone HtpG

Supplementary Table S4. **Upregulated genes expression levels under normal and H_2O_2 stress screened using RNA seq (raw data_log normalization)**

Protein ID	Product	NORMAL		STRESS	
		WT	OT	WT	OT
WP_011244671.1	thioredoxin family protein	11.673	11.291	11.613	11.276
WP_011244428.1	thioredoxin reductase	13.837	13.388	13.683	13.390
	**Redox balance**				
WP_040113194.1	ferredoxin	6.805	6.712	7.040	9.088
WP_011377875.1	ferredoxin-thioredoxin reductase	10.439	10.241	9.977	11.622
WP_011244741.1	catalase peroxidase	10.599	10.836	9.587	12.133
WP_011378008.1	flavodoxin2	11.828	12.292	11.657	13.329
WP_011377579.1	glutathione peroxidase	8.921	8.426	8.562	9.294
WP_011242314.1	flavodoxin	9.770	10.659	10.967	11.462
	**Photosynthesis**				
WP_011378308.1	chlorophyll synthase ChlG	12.196	12.395	11.568	13.634
WP_011243390.1	magnesium protoporphyrin IX methyltransferase	10.042	10.457	10.071	11.357
WP_071818114.1	high light inducible protein	11.013	11.126	10.907	11.926
WP_011244498.1	magnesium-protoporphyrin IX monomethyl ester cyclase	11.386	11.382	11.203	11.975
	**Ion homeostasis**				
WP_011244737.1	potassium channel protein	11.004	11.177	10.546	12.402
WP_011242728.1	ABC transporter ATP-binding protein	11.432	11.746	11.898	12.889
WP_011242460.1	Fe^{3+} ABC transporter substrate-binding protein	10.619	10.737	10.247	11.845
WP_011377873.1	haemolysin-type calcium-binding repeat	11.326	11.097	10.732	12.177
WP_011242780.1	K^+ transporter	12.286	12.425	11.667	13.497
	**Molecular chaperone**				
WP_011378399.1	molecular chaperone DnaJ	10.952	11.021	10.633	12.080
WP_071818122.1	molecular chaperone DnaJ	12.849	12.303	12.558	13.160
WP_011244591.1	molecular chaperone HtpG	13.259	13.155	13.215	13.909
